# Do transportation network companies decrease or increase congestion?

**DOI:** 10.1126/sciadv.aau2670

**Published:** 2019-05-08

**Authors:** Gregory D. Erhardt, Sneha Roy, Drew Cooper, Bhargava Sana, Mei Chen, Joe Castiglione

**Affiliations:** 1 Department of Civil Engineering, University of Kentucky, 161 Raymond Building, Lexington, KY 40506, USA.; 2 San Francisco County Transportation Authority, 1455 Market Street, San Francisco, CA 94103, USA.

## Abstract

This research examines whether transportation network companies (TNCs), such as Uber and Lyft, live up to their stated vision of reducing congestion in major cities. Existing research has produced conflicting results and has been hampered by a lack of data. Using data scraped from the application programming interfaces of two TNCs, combined with observed travel time data, we find that contrary to their vision, TNCs are the biggest contributor to growing traffic congestion in San Francisco. Between 2010 and 2016, weekday vehicle hours of delay increased by 62% compared to 22% in a counterfactual 2016 scenario without TNCs. The findings provide insight into expected changes in major cities as TNCs continue to grow, informing decisions about how to integrate TNCs into the existing transportation system.

## Teaser

Transportation network companies (TNCs), such as Uber and Lyft, are the major driver of increasing traffic congestion in San Francisco.

## INTRODUCTION

Transportation network companies (TNCs) have grown rapidly in recent years. In 2016, TNCs were 15% of all intra-San Francisco vehicle trips, which is 12 times the number of taxi trips (*1*), while in New York in 2016, TNC ridership equaled that of yellow cab and doubled annually between 2014 and 2016 (*2*). TNCs are on-demand ride services where rides are arranged through a mobile app to connect the passenger with a driver, often a private individual driving their personal vehicle (*3*). The current system is commonly viewed as a bridge technology that may be replaced by fleets of self-driving cars if and when that technology is ready (*4*, *5*). TNCs are one form of shared mobility and one form of Mobility-as-a-Service (MaaS). They have been referred to by several names, including ridesourcing, ride-hailing, and e-hail. Ridesourcing is the preferred international standard (*6*), but we refer to TNC throughout this text because it is the legal term used in California, where this study was conducted.

Because they have the potential to reduce the reliance on private cars, the TNCs themselves present a vision of the future in which they reduce traffic congestion and allow roads to be repurposed to other uses (*7*, *8*). There are several mechanisms by which TNCs could reduce congestion. If TNCs are shared concurrently, a service known as ridesplitting, they could reduce traffic if they replace a trip that would otherwise be in a vehicle with fewer occupants. Simulations show that ridesplitting has substantial potential to reduce congestion (*9*). TNCs could induce travelers to shift trips from auto to transit by providing better first- and last-mile connections to regional transit, and there is some evidence to suggest that a small portion of travelers may use TNCs in this way (*10*, *11*). Some have speculated that by providing a convenient alternative to owning a car, TNCs could incentivize people to own fewer cars and, by extension, induce them to shift other trips to transit or non-motorized modes, potentially reducing their total vehicle travel (*12*, *13*).

Competing with these factors are several mechanisms by which TNCs may increase traffic congestion. Deadheading, or out-of-service movement, is the movement of a vehicle with no passenger. TNCs and taxis deadhead to look for fares or reposition before or after a paid trip. Out-of-service travel is estimated at about 50% of TNC vehicle miles traveled (VMT) in New York (*2*) and 20% in San Francisco (*1*). Whether a trip made by TNC adds traffic to the road also depends on which mode would have been used for the trip if TNC was not available. Between 43 and 61% of TNC trips substitute for transit, walk, or bike travel or would not have been made at all (*10*, *11*, *14*, *15*), adding traffic to the road that otherwise would not have been there. TNC pickups and drop-offs (PUDO) contribute to congestion on urban streets by disrupting traffic flow in the curb lane, similar to the congestion effects found in areas that rely heavily on taxis (*16*).

Transportation planners and policy makers are interested in understanding the congestion effects of TNCs as they face decisions about how to regulate TNCs and how to integrate them into the existing transportation system (*17*–*19*). However, studies assessing the net effect of TNCs on congestion have produced mixed results, concluding that TNCs decrease congestion (*20*), TNCs add to VMT or increase congestion (*2*, *14*, *15*), and TNCs “did not drive the recent increase in congestion” (*21*), or have been inconclusive (*10*, *11*). There is a need for further research to adjudicate these differences, but research on the topic has been hampered by a lack of data (*22*, *23*). We enter this debate to address the question: Do TNCs decrease or increase traffic congestion?

We do this for the case of San Francisco while recognizing that the results from a dense and transit-rich city may not translate into many contexts. A data set scraped from the application programming interfaces of the two largest TNCs provides a unique insight into their operations. These data were collected and processed as described by Cooper *et al.* (*23*). We further processed the data to associate TNC volumes, pickups, and drop-offs to each road segment in San Francisco by time of day (TOD). These processed data are included in the Supplementary Materials for use by other researchers.

This study is structured as a before-and-after assessment between 2010 conditions when TNC activity is negligible and 2016 conditions when it is not, focusing on the change in average weekday conditions. We derived measures of roadway conditions in both years from GPS-based speed data licensed from INRIX. We estimated the relationship between the change in TNC activity and the change in roadway travel time, assuming zero TNCs in 2010.

To control for other factors that may also affect congestion over this period, we used San Francisco’s travel demand model, SF-CHAMP, which produces estimates of traffic volumes on all roads in San Francisco and is sensitive to changes in population and demographics, employment, transportation networks, and congestion. Since SF-CHAMP’s initial development (*24*), it has been further enhanced (*25*, *26*), extensively tested (*27*), and successfully applied to analyze policy and infrastructure changes (*28*, *29*). The version of SF-CHAMP used in this study was calibrated to 2010 conditions and does not account for TNCs. This means that when the model is run for current-year inputs, it represents a counterfactual case where TNCs do not exist.

The relationship between demand and traffic speed is nonlinear such that adding vehicles in already congested conditions has a bigger effect than adding them in uncongested conditions. Therefore, it is not just the total VMT change that matters but when and where that change occurs. We conducted our analysis directionally for segments known as traffic messaging channels (TMCs), which average 0.3 miles long. For each year, we aggregated all data to these TMC links and averaged across days to represent average weekday conditions for five TODs. These link-TOD-year combinations are more detailed than past TNC studies, which are either more aggregate (*2*, *13*, *20*, *21*) or based on smaller user surveys (*10*–*12*, *14*, *15*) that cannot be expanded to the link level.

After estimating the relationships between the change in travel times, TNCs, and control variables, we applied the estimated models to evaluate network performance metrics for 2010, 2016, and a counterfactual 2016 scenario with no TNCs. We compared the congestion levels in these two scenarios to evaluate our research question. The discussion section of this paper addresses how our results relate to those of the studies cited above, how the methods compare across these studies, and the limitations of this study, focusing on other changes that may be occurring over this period.

### Observations and hypotheses

**Fig. 1:**
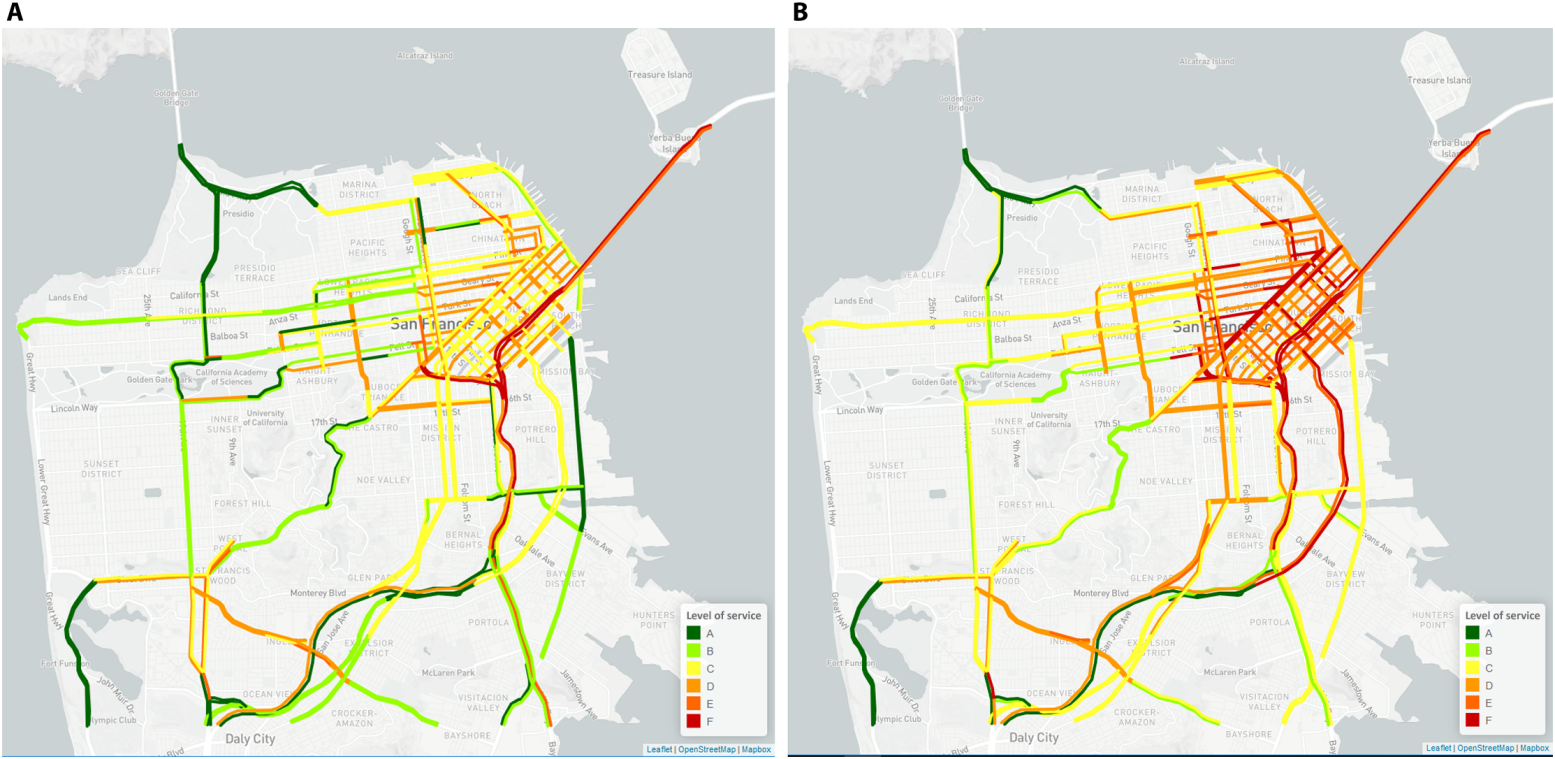
The p.m. peak period roadway level-of-service (LOS) in San Francisco (*30*). (**A**) 2009 conditions; (**B**) 2017 conditions. LOS grades roadways by vehicle delay, from LOS A representing free flow to LOS F representing bumper-to-bumper conditions. Data and an interactive mapping tool are available at congestion.sfcta.org.

Like New York (*2*, *21*), San Francisco has experienced a notable increase in congestion over the past few years (Fig. 1) (*30*). The speed data used in this study confirm this trend, showing that the average speed decreases from 25.6 miles per hour (mph) in 2010 to 22.2 mph in 2016 and that the vehicle hours of delay (VHD) increase by 63% over the same period. Delay is defined as the difference between the congested travel time and the travel time under free-flow conditions.

**Fig. 2:**
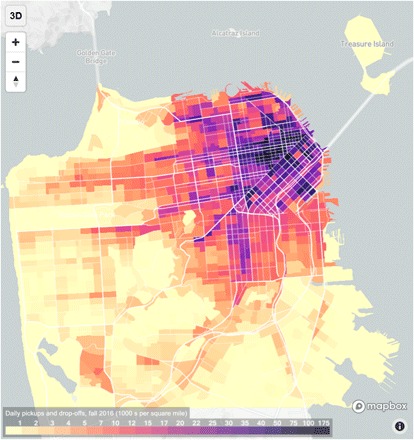
Daily TNC pickups and drop-offs for an average Wednesday in fall 2016 (*1*). Darker colors represent a higher density of TNC activity. Data and an interactive mapping tool are available at tncstoday.sfcta.org.

This change corresponds to the period in which TNCs emerged. Figure 2 shows the distribution of the TNC PUDO for an average Wednesday in fall 2016. The data show that TNCs are concentrated in the downtown area, consistent with findings elsewhere (*11*, *13*), and in the locations where level-of-service deterioration is worst.

Several other changes may also affect congestion. Between 2010 and 2016, San Francisco population grew from 805,000 to 876,000 (*31*) and employment grew from 545,000 to 703,000 (*32*). Important network changes include a rebuild of the Presidio Parkway, the introduction of turn restrictions on Market Street, several “road diets,” and bus improvements (*33*). We account for these changes through SF-CHAMP. In addition, we reviewed a list of active construction projects during the 2016 analysis period to evaluate whether they were associated with disproportionate speed decreases, and did not find that they were.

The data do not show the share of ridesplitting in San Francisco, but it is between 13% and 20% elsewhere (*14*, *15*), with some of those trips carrying no additional passengers (*3*, *15*). Rail ridership grows substantially over this period and bus ridership does not (*34*), consistent with other findings that TNCs may complement rail and compete with bus (*11*, *35*). We do not observe a meaningful change in car ownership, with an average of 1.08 cars per household in 2010 and 1.10 cars per household in 2016 (*36*).

In addition to the 20% of TNC VMT that is out-of-service, 70% of San Francisco TNC drivers live outside the city (*1*). While we do not explicitly track it in this study, the drivers’ commutes into the city may add more VMT to the network. Our data do not provide a direct observation of what TNC users otherwise would have done, so they cannot speak directly to modal substitution. The data do allow us to infer the PUDO locations and associate those locations with specific directional roadways.

Some argue that TNCs have little effect on traffic operations because they occur in the evening when congestion is less severe (*12*, *13*). Our data show not only that 43% of TNC VMT occurs between 6:30 p.m. and 3 a.m. but also that 26% of TNC VMT occurs in the 3-hour a.m. or p.m. peak periods compared to 40% for 4-hour peaks in Boston (*15*).

Given these observations, we suggest that the gap between the background changes predicted by SF-CHAMP and the observed change in travel times is an indicator of TNC impact. Specifically, we hypothesize:

1) If TNCs have no effect on congestion, the background changes should reasonably predict the observed travel time changes.

2) If TNCs decrease congestion, then the observed change in travel time should be better than the background changes would predict.

3) If TNCs increase congestion, then the observed change in travel time should be worse than the background changes would predict. We expect the gap to be biggest for times and locations with high levels of TNC activity.

## MATERIALS AND METHODS

To test these hypotheses, we structured this study as a before-and-after assessment between 2010 conditions where TNC activity is assumed to be negligible and 2016 conditions when they are not. For each year, an estimation data file is compiled with one observation on each road segment and TOD combination. The data represent average weekday conditions in the fall of each year. Fixed-effects panel data models (*37*) are estimated where the dependent variable is a transformed version of the observed travel time, and the descriptive variables include the background traffic levels, TNC volumes, and TNC PUDO. We converted the observed travel times to implied volumes using volume-delay functions (VDFs). This time-implied volume is the model’s dependent variable, and the conversion ensures that it is linearly related to the background and TNC volumes. The fixed-effects models estimate coefficients based on the change between 2010 and 2016 conditions. There is precedent for using both before-and-after analysis and panel data models in transportation analysis, including to study changes in congestion (*38*), TNC growth (*22*), and the effects of new technology (*39*). The estimated coefficients are applied to produce a modeled estimate of 2010 and 2016 network conditions, as well as a 2016 counterfactual scenario that excludes the effect of TNCs.

### Data

The analysis relies on three sources of data: background traffic estimates, TNC data, and speed data. Those data and their processing are described below.

#### Background traffic estimates

To estimate the net effect of TNCs on congestion, it is necessary to control for other factors that are also expected to change congestion levels, including changes to population, employment, and road and transit networks. To control for these changes, this research uses San Francisco’s travel demand model, SF-CHAMP.

SF-CHAMP is an activity-based travel demand microsimulation model that is sensitive to a broad array of conditions that influence travelers’ choices. The model predicts the typical weekday travel patterns for approximately 7.5 million San Francisco Bay Area residents, including choices of vehicle availability, activity participation, destinations, travel modes, and travel times. The simulated travel patterns are sensitive to changes in population and demographics, employment, transportation networks, and congestion. The model incorporates detailed information about demographics and land use, using block, block group, and tract-level geographies, and six broad employment sectors. It also incorporates a detailed representation of the entire Bay Area multimodal transportation system including roadways, transit routes, and non-motorized facilities, as well as information about how these change by TOD. The core behavioral components are based on detailed travel surveys and capture time and cost trade-offs and other factors that influence traveler choices, such as the effects of demographics and the availability and quality of alternatives. The model has been used extensively in practice for almost two decades to evaluate long-range transportation plans, transportation infrastructure investments, pricing policies, and land use development proposals.

SF-CHAMP uses a detailed representation of the road network, including a link for every street and in the city, along with attributes that include length, number of lanes, capacity, turn restrictions, and facility type. The outputs include an estimate of the average weekday traffic volume and congested travel time on each link for each of five TODs: 3 to 6 a.m., 6 to 9 a.m., 9 a.m. to 3:30 p.m., 3:30 to 6:30 p.m., and 6:30 p.m. to 3:00 a.m.

The analysis uses version 5.2.0 of SF-CHAMP, run using 2010 and 2016 inputs. The model runs uses actual inputs, not forecasts, avoiding inaccuracies associated with errors in the inputs. This version of SF-CHAMP was calibrated to 2010 conditions and does not account for TNCs. Normally, this would be a limitation, but in this case, it is beneficial because it means that when the model is run for 2016 population, employment, and network inputs, it represents a counterfactual case where TNCs do not exist.

#### TNC data

Complementing SF-CHAMP are the TNC data, which were collected and processed as described by Cooper *et al.* (*23*). The raw data show the locations and time stamps of out-of-service TNC vehicles collected in 5-s increments for a 6-week period in fall 2016, totaling about 12 terabytes of raw data. When a driver accepts a ride, that vehicle no longer appears in the traces, and after the driver drops off the passenger, the vehicle reappears. This structure allows the analyst to infer that a trip was made between those two points. The point at which the driver disappears from the trace is inferred as the location of a passenger pickup, and the point at which it reappears is inferred as the passenger drop-off location. There is some uncertainty associated with the pickup location because the driver must travel from her current location to the location where the passenger is waiting, but given the density of TNCs in San Francisco, the passenger wait time is usually short. City-wide, the average wait time is 3 min (*40*), and in our experience, it is often 1 to 2 min in the core of the city. Duplicate traces are removed to avoid double-counting drivers who work for both TNCs and vehicles recorded by multiple clients.

The TNC data were further processed for this study in several ways. The out-of-service TNC vehicles were attached to directional SF-CHAMP road links by TOD using a spatial matching process that accounts for the trajectory of points. The in-service TNC volumes were attached to directional road links by assigning each to the shortest path between the inferred PUDO locations, where the shortest path is calculated on the basis of the congested SF-CHAMP networks. Last, the PUDO locations were assigned to directional road links, allowing their effect on congestion to be measured. The end result is a set of SF-CHAMP road networks that include the background traffic volumes and other link attributes, and are annotated with 2016 TNC activity. These are for average weekday conditions, segmented by SF-CHAMP’s five time periods. To the extent that in-service TNC volumes substitute for other auto trips, we expect some overlap between these and the background SF-CHAMP volumes.

#### Speed data

We use archived speed data from INRIX, a commercial vendor, that is available in 5-min increments for each day from 2010 through the present, allowing both the average travel time and reliability metrics to be calculated. Spatially, the data are available directionally for segments known as TMCs, which in San Francisco average about 0.3 miles in length, or about three city blocks. TMCs exclude many local roads but otherwise provide good coverage throughout the city. Links associated with TMCs carry about 70% of the total VMT in San Francisco. This study uses INRIX speed data, at a 5-min temporal resolution, for non-holiday weekdays for the 6-week period in November and December 2016 when TNC data were collected, and for a comparable 6-week period in November and December of 2010. The data are provided for each TMC segment with day and time stamps. A reference speed is also available in the dataset representing speed under uncongested condition.

The speed data depend upon probe vehicles and therefore varies in confidence scores depending upon the TOD and presence of vehicles on each TMC link that provides these data. For the purpose of this study, INRIX speeds pertaining only to the highest confidence score of 30 are used to calculate a reliable estimate for link-resolved travel time. Further, a comprehensive evaluation of the data was conducted, including a comparison to speed data from San Francisco’s Congestion Management Program (*30*). TMC links with unreasonable speeds were excluded from the analysis. For example, a surface street running parallel to a freeway showed unreasonably high speeds, which we suspect is the link picking up probe vehicles from the adjacent freeway. Additional data assurance is performed to identify and exclude data labeled with the wrong travel direction.

Some TMC segments are “filler segments.” Links lying between two stop bars at a traffic signal or unsignalized intersections, links denoting the change in direction of a roadway, etc. are some examples of filler segments. Because these links are extremely short in length (typically, shorter than 0.025 miles) and, more importantly, not representative of a typical roadway segment, they are excluded from the analysis. In total, 23% of TMCs were excluded from the analysis, but these TMCs account for less than 4% of the total TMC road length.

To incorporate the predicted volume obtained from the SF-CHAMP model, as well as normalizing the growth in background traffic attributable to the typical non-TNC factors, it is required to create an association between the TMC network and the SF-CHAMP network. The remaining TMC links are associated with the corresponding SF-CHAMP links. In most cases, SF-CHAMP links aggregate to TMC links. In instances when a CHAMP segment is longer than a TMC segment, multiple TMC segments were merged together to form one composite TMC segment and correspond to the said CHAMP segment. In a few cases, such as in some of the more complex freeway interchanges, a clean correspondence could not be identified between the SF-CHAMP links and the TMC links. Those cases were excluded from the analysis.

The 5-min speed data were aggregated to average weekday measures for each of the five SF-CHAMP time periods. During this aggregation, several speed metrics were calculated, including the mean, the standard deviation, the 5th percentile, and the 20th percentile. The highest observed average hourly speed on each TMC link over the observation period was assigned as the free-flow speed for that link. Examination shows that the free-flow speed on a segment remained largely unchanged between 2010 and 2016.

#### Merging the data

The data were merged such that TMC links serve as the common spatial units for the remainder of the analysis. When the data were aggregated from the SF-CHAMP links to the TMC links, the link attributes were also aggregated. Volumes and capacities were combined using a length-weighted average. There are two measures of distance: one from the SF-CHAMP links and one from the TMC links. The SF-CHAMP links are more spatially accurate, so the sum of the SF-CHAMP link length was used as the primary measure of length in the combined data set. In the event where multiple TMC segments need to be aggregated, the space mean speed was estimated by dividing the combined TMC length by the sum of travel time across all TMCs. The speed was then applied to the length of the combined SF-CHAMP links.

All of this was done for both 2010 and 2016 scenarios. The 2010 and 2016 data were matched for each TMC segment, and if there were missing data in one or the other, both records were dropped. This can happen, particularly in the 3 to 6 a.m. time period, if there are insufficient probe vehicles to achieve the highest confidence score in the INRIX data. The end result is a matched panel with 2010 and 2016 for a total of 7082 TMC link–TOD combinations. This corresponds to 1450 TMC links with up to five TODs each. The resulting estimation files are included in the Supplementary Materials as part of data S3.

## METHODS

To estimate the effect of TNCs, we used a fixed-effects panel data regression model (*37*). The fixed-effects standardize the link-dependent unexplained constancy or variance that might affect the regressed variable. Some examples of link-specific characteristics are location of links near high foot traffic, recreational areas, and special roadway geometry. The temporal unit used by the panel is 2, warranted by the before-after nature of the study. Each data point in the dataset is a unique combination of a TMC, TOD, and observation year. Because there are only two points in time, this is equivalent to estimating an ordinary least squares (OLS) model on the change on each TMC for each TOD.

### Converting travel time to implied volume

A challenge in estimating these models is that they assume a linear relationship between the dependent variable and the regressors, but the relationship between volume and travel time is nonlinear. To deal with this, the VDFs from SF-CHAMP were used to convert the observed travel times into implied passenger car equivalent (PCE) volumes. They take the form

$$T=T_{0}(1+\alpha {\left(\frac{V}{C}\right)}^{\beta })$$ (1)

where *T* is the congested travel time, *T*_0_ is the free-flow travel time, *V* is the traffic volume in PCEs, *C* is the link capacity, and α and β are calibrated parameters. Solving for *V*, we get

$$V_{I}=C{\left(\frac{\frac{T}{T_{0}}-1}{\alpha }\right)}^{1/\beta }$$ (2)

where the subscript on *V*_I_ is used to designate a time-implied volume, as derived from the travel times. The panel models use *V*_I_ as their dependent variable. It is in units of PCEs, so it is linearly related to the volume measures in the descriptive variables.

The analysis was conducted for five multi-hour time periods, so it is important that all volumes and capacities are either hourly or for the period as a whole. Here, we defined them for the period as a whole and scaled the hourly capacities to the period total using the same peak hour factors that were used by SF-CHAMP.

### Congestion effects of PUDO

In considering the effect of TNC PUDO on congestion, it is useful to consider other scenarios in which a vehicle movement has an effect on congestion beyond simply driving on the roadway. Several examples where this occurs include taxis (*16*), delivery trucks (*41*), and movements into or out of on-street parking spaces (*42*, *43*). Wijayaratna (*44*) provides a useful method for considering the congestion effect of on-street parking that follows the capacity adjustment approach used frequently in the Highway Capacity Manual (*45*). The approach scales the capacity of the road lane adjacent to the on-street parking based on the share of time that the lane is blocked. To model the effect of TNC PUDO, we took a similar approach, but defined the PUDO effect in PCEs so that it was in the same units as our dependent variable, and expressed the effect as

$$\beta_{\text{AvgDur}}*\frac{\text{PUDO}*\text{PHF}}{3600}*\frac{C}{L}$$ (3)

where PUDO is the number of PUDO in the period, PHF is the peak hour factor to convert the PUDO to an hourly value, *C* is the capacity of the link, *L* is the number of lanes, and β_AvgDur_ is an estimated model parameter. For simplicity, we expressed this term, excluding the estimated coefficient, as *V*_AvgDur_. β_AvgDur_ can be interpreted as the average duration that each PUDO blocks or disturbs traffic in the curb lane. In congested conditions, this can be longer than the duration of the stop itself, because it can take some time for a queue to dissipate if it builds up behind a stopped vehicle and for traffic to recover to its pre-PUDO condition. β_AvgDur_ can also be shorter than the actual duration of a stop if there is some probability that the stopping vehicle can pull out of traffic or if volumes are low enough that the probability of a vehicle arriving behind the stopped vehicle is low.

### Model estimation

To estimate the effect of other factors on the change in implied volume, we used a fixed-effects panel data regression model (*37*). The fixed-effects standardize the link-dependent unexplained constancy or variance that might affect the regressed variable. Some examples of link-specific characteristics are location of links near high foot traffic, recreational areas, and special roadway geometry. Because these characteristics do not change between 2010 and 2016, their influence is absorbed into the fixed effect, preventing them from biasing the other parameter estimates. The temporal unit used by the panel is 2, warranted by the before-after nature of the study. Each data point in the dataset is a unique combination of a TMC, TOD, and observation year. Because there are only two points in time, this is equivalent to estimating an OLS model on the change on each TMC for each TOD. The estimated model can be expressed as

VI:i,t=β1VSF−CHAMP:i,t+β2VTNC:i,t+β3FTMajArt:i*VAvgDur:i,t+β4FTMinArt:i*VAvgDur:i,t+β5PRESIDIOi,t*VI:i,2010+FEi+εi,t (4)

where the entities *i* are TMC links by TOD and the time periods *t* are either 2010 or 2016, and each is used to index the remaining variables. *V*_I:*i*,*t*_ is the time-implied volume. *V*_SF − CHAMP:*i*,*t*_ is the volume predicted by SF-CHAMP in PCE, giving some additional weight to trucks and buses. *V*_AvgDur:*i*,*t*_ is the average duration variable, as defined above. FT_MajArt:*i*_ is a binary facility type flag indicating whether or not the link is a major arterial, and FT_MinArt:*i*_ is a binary facility type flag indicating whether or not the link is a minor arterial. These facility type flags do not change between the two years. PRESIDIO_*i*,*t*_ is a binary flag identifying links on the Presidio Parkway and Veterans Boulevard, where there was major construction in 2010 but not in 2016. PRESIDIO_*i*,*t*_ is defined to be zero in 2010 and one in 2016 such that the effect of a change can be estimated. *V*_I:*i*,2010_ is the time-implied volume in period 1 (2010), which allows the effect of the construction change to be proportional to the starting volume on the link, as opposed to additive and the same on every link. FE*_i_* is the fixed-effect, which is effectively a constant on each entity, and ε_*i*, *t*_ is a random error term. In this specification, the Presidio flag (PRESIDIO_*i*, *t*_) and the TNC terms (*V*_TNC:*i*,*t*_, *V*_AvgDur:*i*,*t*_) are zero in 2010, so the 2010 time-implied volume is simply a function of the SF-CHAMP volume plus the fixed effect and an error term.

A number of variations on this specification were attempted before arriving at the preferred model. For example, specifications were tested that split the TNC volume into separate in-service and out-of-service volumes or segmented the PUDO coefficients in different dimensions. One notable variation relates to our hypothesis that TNCs have no effect on traffic congestion. If this were true, we would expect the change in background volume alone to reasonably predict the change in time-implied volume (*V*_I_). Estimating such a model reveals that the background volume is highly correlated with *V*_I_, with a coefficient of 1.78. This suggests that time-implied volumes are increasing by 78% more than SF-CHAMP would predict. It appears that the employment, population, and network changes do not fully describe the congestion changes observed during this period, and more terms are needed to do so.

### Model application

After the model was estimated, it was applied to all links to predict the *V*_I:i,t_ for 2010 and 2016. It was also applied to predict a 2016 counterfactual scenario with no TNCs by setting *V*_TNC:i,2016_ and *V*_AvgDur:i,2016_ to zero and otherwise applying the model to 2016 data. These predicted PCEs were then used to calculate the travel times using the VDFs (Eq. 1).

The non-PCE volume on each link is calculated as

$$V_{i,t}=V_{\text{SF}‐\text{CHAMP}:i,t}+\beta_{2}V_{\text{TNC}:i,t}$$ (5)

where *V*_*i*,*t*_ is the traffic volume in units of vehicles instead of PCEs and *V*_SF − CHAMP:*i*,*t*_ is the SF-CHAMP volume. β_1_ is excluded such that we count the full SF-CHAMP traffic volume, not their estimated effect on speed. The inclusion of β_2_ (which is less than one) accounts for the partial overlap between the TNC volumes and the background volumes. These volumes were combined with the link lengths to calculate VMT, and combined with travel times to calculate vehicle hours traveled (VHT) and VHD. The average speed is calculated as VMT/VHT. The same volumes were used in combination with observed travel times to calculate observed VHT, VHD, and average speed. In addition, a set of reliability metrics was calculated as described below.

### Travel time reliability metric

This study uses planning time index 80 (PTI80) as the measure of travel time reliability. It is defined as

$$\text{PTI}80=\frac{T_{80}}{T_{0}}$$ (6)

where *T*_80_ is the 80th percentile travel time and *T*_0_ is the free-flow travel time. A PTI80 value of 1.5 means that for a 30-min trip in light traffic, 45 min should be planned to ensure on-time arrival 80% of the time.

PTI80 can be calculated directly using measured travel times, or estimated as a function of the travel time index (TTI) (*46*), which is the ratio between the average travel time and the free-flow travel time. The estimated relationship for each observation *i* takes the form

$$\text{PTI}80_{i}=\gamma_{1}{{\mathrm{TTI}}_{i}}^{\gamma_{2}}$$ (7)

where γ_1_ and γ_2_ are estimated model parameters. These parameters were estimated for this study from the observed travel time data from both 2010 and 2016, with one observation for each TMC, TOD, and year combination. The relationships are specific to each facility type. Table 1 shows the results of that estimation. PTI80 was calculated for each TMC link, TOD, and year combination, and was aggregated to the network level using a VMT-weighted average.

**Table 1:** Estimated relationships between PTI80 and TTI.

**Facility type**	**γ**_**1**_	**γ**_**2**_	***R***^**2**^
Freeways and expressways	1.029	1.498	0.831
Arterials	1.101	1.361	0.862
Collectors and locals	1.131	1.440	0.762

## RESULTS

Table 2 shows our model estimation results from the fixed-effects models. The SF-CHAMP background volume parameter estimate is 0.92, not significantly different than 1. This is logical, because we expect that each vehicle added in background traffic should have an effect on congestion of adding one vehicle to the implied volume. The Presidio Parkway scaling factor accounts for major construction that was underway on those links in 2010 but not 2016, and is equivalent to reducing the 2010 implied traffic volume by 36%.

**Table 2:** Fixed-effects panel estimation results with TNC variables.

**Parameter estimates**			
**Variable**	**Parameter**	**Standard error**	***T*-statistic**
SF-CHAMP background volume	0.9172	0.0541	16.952
Presidio Parkway scaling factor	−0.3648	0.0189	−19.327
TNC volume	0.6864	0.0720	9.5387
Average impact duration of TNC PUDO on major arterials (s)	144.75	7.7195	18.751
Average impact duration of TNC PUDO on minor arterials (s)	79.486	12.114	6.5617
**Model statistics**			
Number of entities	7081		
Number of time periods	2		
*R*^2^ between groups	0.5819		
*R*^2^ within groups	0.2985		

We include two measures of time- and location-specific TNC activity. The TNC volume parameter measures the net effect of TNCs. If TNCs purely substitute for other car trips, the estimated TNC parameter should be zero as they substitute for other vehicles already counted in the background volumes. Negative values would be consistent with TNCs reducing traffic, while a value of positive 1 would be consistent with TNCs purely adding to background traffic. The estimated coefficient of 0.69 can be interpreted as an addition of one TNC vehicle, partially offset by a subtraction of 0.31 non-TNC vehicles.

The PUDO parameters represent the average number of seconds that a pickup or drop-off disrupts traffic in the curb lane. Locally collected data show that the average time needed for a passenger to board or alight from passenger vehicles such as TNCs and taxis is about 1 min. The higher average impact durations estimated in these models suggest that the traffic disruption persists after the stopped vehicle departs because additional time is needed for traffic flow to recover to its pre-PUDO condition.

We applied the estimated model to assess network-wide performance metrics for three scenarios:

1) 2010: reflecting observed 2010 conditions, when no TNCs were present;

2) 2016 no TNC: represents a counterfactual scenario of what 2016 conditions would be if there were no TNCs;

3) 2016 with TNC: the full application of the model to 2016 conditions.

Table 3 presents network performance metrics for these three scenarios. VMT grows by 13% between 2010 and 2016, with almost half of the VMT increase attributable to TNCs. We calculate VHT, VHD, and average speed using both modeled travel times and, where available, observed travel times. Without TNCs, VHT would be 12% higher in 2016 than in 2010, VHD would be 22% higher, and average speed would be 4% lower. With TNCs, VHT is 30% higher, VHD is 62% higher, and speeds are 13% lower.

**Table 3:** Network performance metrics.

**Scenario**	**Network performance metrics**								
	**VMT**	**Based on modeled travel time**			**Based on observed travel time**				
		**VHT**	**VHD**	**Average speed (mph)**	**PTI80**	**VHT**	**VHD**	**Average speed (mph)**	**PTI80**
2010	4,923,449	205,391	64,863	24.0	1.83	204,686	64,158	24.1	1.83
2016 no TNC	5,280,836	230,642	79,449	22.9	1.94	N/A	N/A	N/A	N/A
2016 with TNC	5,559,412	266,393	105,377	20.9	2.12	269,151	108,134	20.7	2.21
**Scenario**	**Percent change from 2010**								
	**VMT**	**Based on modeled travel time**				**Based on observed travel time**			
		**VHT**	**VHD**	**Average speed (mph)**	**PTI80**	**VHT**	**VHD**	**Average speed (mph)**	**PTI80**
2010	0%	0%	0%	0%	0%	0%	0%	0%	0%
2016 no TNC	7%	12%	22%	−4%	6%	N/A	N/A	N/A	N/A
2016 with TNC	13%	30%	62%	−13%	15%	31%	69%	−14%	21%

In addition, travel time is becoming less reliable, as measured by PTI80. PTI80 is the ratio between the 80th percentile travel time and the free-flow travel time. It is a measure of the day-to-day variability of travel time. A PTI80 value of 1.8 means that for a 10-min trip in uncongested condition, 18 min should be planned to ensure on-time arrival 80% of the time. Between 2010 and 2016, PTI80 increases by 15% with TNCs or by 6% without TNCs.

**Fig. 3:**
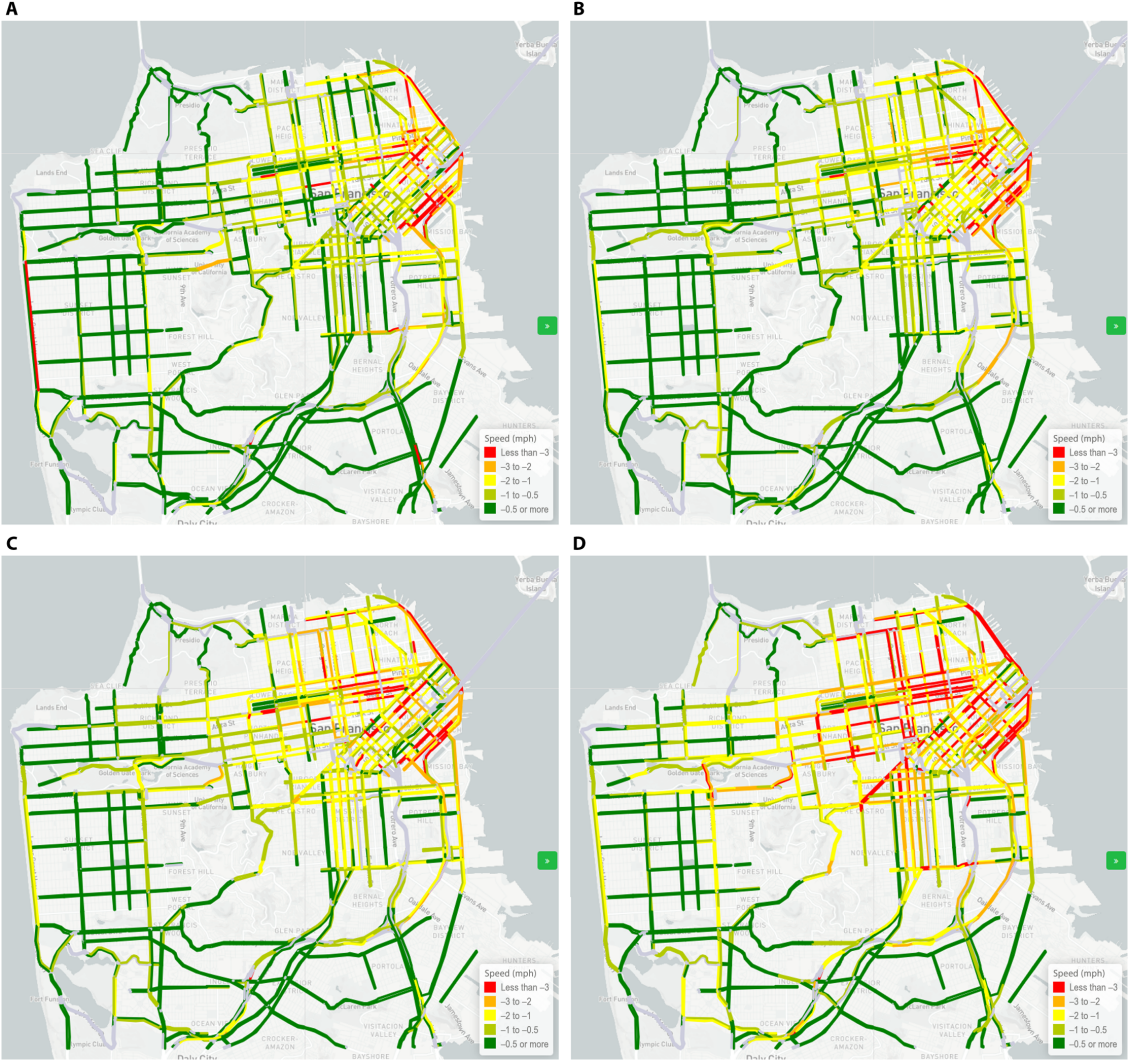
Speed (mph) difference between 2016 scenario with TNCs and a counterfactual 2016 scenario without TNCs. Data represent four times of day: (**A**) 6 to 9 a.m.; (**B**) 9 a.m. to 3:30 p.m.; (**C**) 3:30 to 6:30 p.m.; and (**D**) 6:30 p.m. to 3:00 a.m. Data are provided in the Supplementary Materials.

The distribution of congestion effects is not uniform throughout the network or throughout the day. Figure 3 maps the speed difference between the TNC scenario and the no-TNC counterfactual for four TODs. TNCs have a larger effect on congestion in the downtown area and on arterial roadways. TNCs have a disproportionately large effect on evening congestion, but they also increase congestion in the peak periods: a 48 to 52% increase in VHD in the a.m. and p.m. periods with TNCs versus an 18 to 23% increase for the no-TNC counterfactual. Additional tables showing TNC effect by TOD, facility type, and area type are in the Supplementary Materials.

## DISCUSSION

Our results show higher VMT and more congestion in the 2016 TNC scenario than in the no-TNC counterfactual. These results are consistent with the subset of TNC rider surveys that were able to draw a conclusion about the net VMT effect of TNCs (*14*, *15*). Both of these studies were based on intercept surveys where the TNC driver asks the passenger what mode they would have chosen had they not used a TNC. Our results are also consistent with the most recent findings in New York that TNCs add VMT and increase congestion (*2*). This study is the most similar to our own, in that it is based on an assessment of data on e-hail trips, which are required to be reported to the city’s Taxi and Limousine Commission. Our results provide complementary evidence to the subset of surveys that were inconclusive regarding the net effect of TNCs on VMT (*10*, *11*). The first such study is based on a survey distributed at TNC hot spots in San Francisco during May and June 2014 (*10*). This study is useful because it provided an early view into the TNC market, although it is recognized both that the market has evolved over the intervening 2.5 years and that this intercept method provides for a sample that is spatially tied to trips at those specific hot spots. The second is based on a survey conducted from 2014 to 2016 in seven U.S. cities (*11*). It explicitly explores the question of whether TNC users reduce their vehicle holdings, and whether that results in reduced vehicle travel, finding “Those who have reduced the number of cars they own and the average number of miles they drive personally have substituted those trips with increased ride-hailing use. Net VMT changes are unknown.” Our findings differ from the conclusions of several other studies (*9*, *12*, *13*, *20*, *21*). The relationship between our findings and those of other studies is discussed below.

A study by Li *et al*. finds “reasonable evidence that the entry of Uber significantly decreases traffic congestion in the urban areas of the U.S.” (*20*). This study estimates models of the change in annual congestion in metropolitan areas from 1982 to 2014, as measured by the Urban Mobility Report (*47*). It introduces a binary variable into the model based on the year of Uber’s entry into each market and uses the negative coefficient estimate as the basis for their conclusion. There are two issues with this approach. First, it does not reflect spatial detail in the distribution of TNCs, which are heavily concentrated in downtown areas, so the aggregate nature of the study may obscure the underlying effect. Second, it does not capture the quantity of TNC use, which varies between cities and continues to grow after entering a market. Our study does better on both accounts.

The City of New York (*21*) used New York’s travel demand model to develop 2010 and 2020 VMT estimates and examined e-dispatch trip records in comparison to those total VMT estimates. They based their conclusion that TNCs did not drive the recent increase in congestion on a projection that TNCs largely substitute for yellow taxi trips and on a lack of evidence for congestion effects associated with PUDO. Our results show that, at least in San Francisco, substitution for taxis and cars only offsets a portion of the TNC volume, and they provide evidence of a PUDO effect.

Simulations, such as the “Portugal Study,” showing large benefits from ridesplitting assume full participation and centralized optimization (*9*). These assumptions do not reflect the way in which TNCs operate today. While our data do not include vehicle occupancy, other survey data show a modest share of ridesplitting (*14*, *15*), and our results suggest that it is not sufficient to offset the ways in which TNCs add to congestion. Such simulations can be useful in establishing the positive potential of ridesplitting if such a system were effectively managed to achieve socially desirable outcomes, but do not imply that TNCs will achieve those outcomes on their own.

Two notable studies by Feigon and Murphy (*12*, *13*) promote the idea of TNCs as a complement to public transit. These studies base their conclusions primarily on data summaries generated from surveys of shared mobility users. Feigon and Murphy conclude that because TNC use is high in the evening and weekend periods when transit service is less frequent, TNCs largely complement public transit and enhance urban mobility (*12*, *13*). However, their own data show (*13*), and ours confirm, that TNC use is also high during the peak periods when congestion is worst and transit service is frequent. Feigon and Murphy find that a greater use of shared modes is associated with more frequent transit use (*12*). However, this finding should not be taken to imply a directional relationship, as it could be that frequent transit users are likely to switch some trips to TNC, adding traffic to the roads. Feigon and Murphy also note that TNC use is associated with decreases in respondents’ vehicle ownership and private vehicle trips (*13*). While this may be true of specific users, we do not observe aggregate changes in vehicle ownership in San Francisco between 2010 and 2016. Further, this finding only accounts for the subtraction of private vehicle trips, not the addition of TNC vehicle trips. Our results indicate that the net effect of TNCs is to add more vehicles to the road.

Some limitations of this study are worth noting. First, the analysis relies on VDFs that are limited in their ability to capture the underlying complexity of traffic flow (*48*). They should be viewed as a means of understanding the aggregate relationships observed in the data, not of the expected operations at a specific location.

Second, while the predicted background traffic changes account for several important control variables, there remains a risk that our results are confounded by another factor. For example, our analysis controls for demographic and socioeconomic changes over this period, but like all travel models, SF-CHAMP assumes that the relationship between those inputs and the resulting travel behavior remains stable. If there are major behavioral changes over this period, it could affect the result. For example, representatives from Uber argue that growing congestion may instead be due to growing freight deliveries or increased tourism (*49*). We discuss each of these possibilities.

Regarding freight deliveries, our analysis reflects growth in truck travel associated with growing employment, but it does not account for structural changes such as a large shift from in-person to online shopping. Such a shift could increase delivery truck volumes but decrease personal shopping trips (*50*). The net effect of this trade-off is not clear and depends on factors such as how efficiently the delivery vehicle can chain multiple deliveries together, what TOD the different trips would occur, and whether the deliveries are to commercial locations in the downtown area or to less congested residential areas. Unfortunately, we lack the commercial vehicle data necessary to evaluate that effect.

Regarding tourism, SF-CHAMP does include a visitor model, with visitor travel representing 4.5% of intra-San Francisco person trips in 2010. The visitor model is influenced primarily by the number of hotel rooms in the city, which have not increased significantly over this period. Data from the San Francisco Council on Economic Development (*51*) show that the number of visitors to San Francisco grew by 22% between 2010 and 2016, with some of those visitors staying in lodging options such as Airbnb, which are not reflected in our visitor model. When this growth is applied to the base of 2010 visitor trips, it might generate up to 1% more intra-San Francisco person trips, beyond what is already included in the background growth. However, these visitor trips only add to congestion if they are in a vehicle, with transit, walk, and TNC being the most commonly used modes among visitors. Thus, due to the growth in tourism, the total vehicle trips in 2016 may be a fraction of a percent higher than we estimate in the background traffic volumes. For comparison, TNCs are 15% of intra-San Francisco vehicle trips in 2016.

As we consider the possibility of other uncontrolled factors, it is worth keeping in mind a few aspects of our research. To have an effect, any uncontrolled factors must be different between 2010 and 2016. Also, our estimation results show that congestion is growing more than expected specifically on the links and in time periods with high levels of TNC activity. The most problematic factors would be those that are spatially and temporally correlated with TNCs, occurring on those same links in the same time periods.

Third, the analysis presented here is specific to a single city with a dense urban core and a rich transit system. The data show that TNC use is heavily concentrated in the densest portion of that city, consistent with evidence from other cities (*13*). While we may expect similar results in other comparable cities, further research is needed to confirm that expectation. The effects of TNCs may be quite different in smaller cities, in less dense areas, or in places with very different populations or regulatory environments.

Several extensions would complement this research: better understanding the contributors to background growth, assessing the TNC effect on transit ridership, and considering how worsening congestion and travel time reliability affect transit operations. Last, the study should be repeated elsewhere to understand how the results vary in cities of different sizes and compositions.

## CONCLUSIONS

This study examines the effect of TNCs on traffic congestion and reliability in San Francisco. It is intended to adjudicate between competing arguments about whether TNCs decrease or increase congestion.

The results show that the observed changes in travel time are worse than the background changes would predict. The estimated TNC volume and PUDO coefficients show that travel times get worse on roads with more TNC activity than on roads with less TNC activity after controlling for background traffic changes. This result supports the hypothesis that TNCs increase congestion, at least in San Francisco.

The results show some substitution between TNCs and other car trips, but that most TNC trips are adding new cars to the road. The estimated models show that TNC vehicles stopping at the curb to pick up or drop off passengers have a notable disruptive effect on traffic flow, especially on major arterials.

The model is applied to estimate network-wide conditions for 2016 and for a counterfactual scenario that estimates what conditions would be in 2016 if there were no TNCs. Both are compared to a 2010 baseline, before TNCs. VMT, VHT, and VHD increase by 13, 30, and 62%, respectively, from 2010 to 2016. Without TNCs, those same metrics would have increased by 7, 12, and 22%. Average speeds decrease by 13%, compared to a 4% decrease without TNCs. TNCs are associated with worsening travel time reliability, thus requiring travelers to further buffer their travel times if they wish to consistently arrive on time. These results lead us to conclude that TNCs are the biggest factor driving the rapid growth of congestion and deterioration of travel time reliability in San Francisco between 2010 and 2016, exceeding the combined effects of population growth, employment growth, and network changes.

These findings are of interest to transportation planners, to policy makers, and to the general public in San Francisco and other large cities. It is in the public interest that decisions about the regulation of TNCs, the allocation of curb space and right-of-way, and the integration of new mobility services with existing transit operations be based on independent and peer-reviewed analysis as presented here.

## Supplementary Material

http://advances.sciencemag.org/cgi/content/full/5/5/eaau2670/DC1

Download PDF

Data S1

Data S2

Data S3

Data S4

## SUPPLEMENTARY MATERIALS

Supplementary material for this article is available at http://advances.sciencemag.org/cgi/content/full/5/5/eaau2670/DC1 Supplementary Text Fig. S1. Area type map on SF-CHAMP links. Table S1. Fixed-effects panel model estimation results only accounting for background traffic. Table S2. Network performance metrics by TOD. Table S3. Network performance metrics by area type. Table S4. Network performance metrics by facility type. Data S1. Supporting data for Fig. 1. Data S2. Supporting data for Fig. 2. Data S3. Model estimation files. Data S4. Model application results and supporting data for Fig. 3.

## References

[1] *San Francisco County Transportation Authority, TNCs Today: A Profile of San Francisco Transportation Network Company Activity* (San Francisco County Transportation Authority, 2017).

[2] B. Schaller, *Unsustainable? The Growth of App-Based Ride Services and Traffic, Travel and the Future of New York City* (Schaller Consulting, 2017); http://www.schallerconsult.com/rideservices/unsustainable.pdf.

[3] Transportation Research Board, “Between public and private mobility: Examining the rise of technology-enabled transportation services” (TRB Special Report 319, Transportation Research Board, 2016).

[4] Zmud J. P., Sener I. N., Towards an understanding of the travel behavior impact of autonomous vehicles. Transp. Res. Procedia 25, 2500–2519 (2017).

[5] Fagnant D. J., Kockelman K. M., Dynamic ride-sharing and fleet sizing for a system of shared autonomous vehicles in Austin, Texas. Transportation 45, 143–158 (2018).

[6] SAE International, *Taxonomy and Definitions for Terms Related to Shared Mobility and Enabling Technologies* (SAE International, 2018); http://standards.sae.org/J3163_201809.

[7] Uber Newsroom; https://www.uber.com/newsroom/company-info/.

[8] J. Zimmer, The Third Transportation Revolution (2016); https://medium.com/@johnzimmer/the-third-transportation-revolution-27860f05fa91.

[9] Martinez L. M., Viegas J. M., Assessing the impacts of deploying a shared self-driving urban mobility system: An agent-based model applied to the city of Lisbon, Portugal. Int. J. Transp. Sci. Technol. 6, 13–27 (2017).

[10] Rayle L., Dai D., Chan N., Cervero R., Shaheen S., Just a better taxi? A survey-based comparison of taxis, transit, and ridesourcing services in San Francisco. Transp. Policy 45, 168–178 (2016).

[11] R. R. Clewlow, G. S. Mishra, “Disruptive transportation: The adoption, utilization, and impacts of ride-hailing in the United States” (Research Report UCD-ITS-RR-17-07, UC Davis, 2017).

[12] S. Feigon, C. Murphy, “Shared mobility and the transformation of public transit” (Transit Cooperative Research Program Report 188, Transportation Research Board, 2016).

[13] S. Feigon, C. Murphy, “Broadening understanding of the interplay between public transit, shared mobility, and personal automobiles” (Transit Cooperative Research Program Report 195, Transportation Research Board, 2018).

[14] A. Henao, “Impacts of ridesourcing—Lyft and Uber—on transportation including VMT, mode replacement, parking, and travel behavior,” thesis, University of Colorado at Denver (2017).

[15] S. Gehrke, A. Felix, T. Reardon, *Fare Choices Survey of Ride-Hailing Passengers in Metro Boston* (Metropolitan Area Planning Council, 2018).

[16] Golias I., Karlaftis M. G., The taxi market in Athens, Greece, and its impact on urban traffic. Transp. Q. 55, 63–71 (2001).

[17] J. Kuhr, C. R. Bhat, J. Duthie, N. Ruiz, Ridesharing & public-private partnerships: Current issues, a proposed framework and benefits, in *Transportation Research Board 96th Annual Meeting*, Washington, DC, 8 to 12 January 2017.

[18] Moran M., Lasley P., Legislating transportation network companies. Transp. Res. Rec. 2650, 163–171 (2017).

[19] A. Cohen, S. Shaheen, “Planning for shared mobility” (PAS Report 583, American Planning Association, 2016), p. 110.

[20] Z. Li, Y. Hong, Z. Zhang, An empirical analysis of on-demand ride-sharing and traffic congestion, in *International Conference on Information Systems (ICIS’16)*, Dublin, Ireland, 11 to 14 December 2016.

[21] City of New York, Office of the Mayor, *For Hire Vehicle Transportation Study* (City of New York, Office of the Mayor, 2016).

[22] R. Gerte, K. C. Konduri, N. Eluru, Is there a limit to adoption of dynamic ridesharing systems? Evidence from analysis of Uber demand data from New York City, in *TRB Annual Meeting*, Washington, DC, 2018.

[23] D. Cooper, J. Castiglione, A. Mislove, C. Wilson, Profiling TNC activity using big data, in *TRB Annual Meeting*, Washington, DC, 2018.

[24] Jonnalagadda N., Freedman J., Davidson W. A., Hunt J. D., Development of microsimulation activity-based model for San Francisco: Destination and mode choice models. Transp. Res. Rec. 1777, 25–35 (2001).

[25] G. Erhardt, B. Charlton, J. Freedman, J. Castiglione, M. Bradley, Enhancement and application of an activity-based travel model for congestion pricing, in *Innovations in Travel Modeling Conference*, Portland, OR, 22 to 24 June 2008.

[26] Zorn L., Sall E., Wu D., Incorporating crowding into the San Francisco activity-based travel model. Transportation 39, 755–771 (2012).

[27] M. Outwater, B. Charlton, The San Francisco model in practice: Validation, testing, and application, in *Innovations in Travel Demand Modeling, Volume 2: Papers*, Austin, TX, 21 to 23 May 2006.

[28] Castiglione J., Hiatt R., Chang T., Charlton B., Application of travel demand microsimulation model for equity analysis. Transp. Res. Rec. 1977, 35–42 (2006).

[29] Brisson E. M., Sall E., Ang-Olson J., Achieving goals of San Francisco, California, for greenhouse gas reductions in transportation sector: What would it take? Transp. Res. Rec. 2287, 89–97 (2012).

[30] San Francisco County Transportation Authority, *2017 Congestion Management Program* (San Francisco County Transportation Authority, 2017).

[31] U.S. Census Bureau, Annual Estimates of the Resident Population Table PEPANNRES.

[32] U.S. Bureau of Labor Statistics, Quarterly Census of Employment and Wages.

[33] SFMTA, Projects; https://www.sfmta.com/projects.

[34] G. D. Erhardt, “Fusion of large continuously collected data sources: Understanding travel demand trends and measuring transport project impacts,” thesis, University College London (2016).

[35] R. Mucci, “Transportation network companies: Influencers of transit ridership trends,” thesis, University of Kentucky (2017).

[36] U.S. Census Bureau, *American Community Survey 2010 and 2016 1-Year Estimates* (U.S. Census Bureau, 2016).

[37] W. H. Greene, *Econometric Analysis* (Prentice Hall, ed. 5, 2003).

[38] Hanna R., Kreindler G., Olken B. A., Citywide effects of high-occupancy vehicle restrictions: Evidence from “three-in-one” in Jakarta. Science 357, 89–93 (2017). 28684524 10.1126/science.aan2747

[39] Tang L., Thakuriah P., Ridership effects of real-time bus information system: A case study in the city of Chicago. Transp. Res. Part C Emerg. Technol. 22, 146–161 (2012).

[40] San Francisco County Transportation Authority, “Emerging mobility evaluation report” (Draft Report, San Francisco County Transportation Authority, 2018); http://www.sfcta.org/sites/default/files/Emerging%20Mobility%20Evaluation%20Report%2004242018.pdf).

[41] Chiabaut N., Investigating impacts of pickup-delivery maneuvers on traffic flow dynamics. Transp. Res. Procedia 6, 351–364 (2015).

[42] Yousif S., A study into on-street parking: Effects on traffic congestion. Traffic Eng. Control 40, 424–427 (1999).

[43] Biswas S., Chandra S., Ghosh I., Effects of on-street parking in urban context: A critical review. Transp. Dev. Econ. 3, 10 (2017).

[44] S. Wijayaratna, Impacts of on-street parking on road capacity, in *Australasian Transport Research Forum*, Sydney, Australia, 30 September to 2 October 2015, pp. 1–15.

[45] Transportation Research Board, *Highway Capacity Manual: HCM 2010* (Transportation Research Board, ed. 5, 2010).

[46] Cambridge Systematics Inc., Texas A&M Transportation Institute, University of Washington, Dowling Associates, Street Smarts, H. Levinson, H. Rakha, “Analytical procedures for determining the impacts of reliability mitigation strategies” (SHRP 2 Report S2-L03-RR-1, Transportation Research Board, 2012).

[47] D. Schrank, B. Eisele, T. Lomax, J. Bak, *2015 Urban Mobility Scorecard* (Texas A&M Transportation Institute and Inrix Inc., 2015); https://mobility.tamu.edu/ums/.

[48] Y.-C. Chiu, J. Bottom, M. Mahut, A. Paz, R. Balakrishna, T. Waller, J. Hicks, *Dynamic Traffic Assignment: A Primer* (Transportation Research Circular E-C153, 2011).

[49] C. Said, “Uber, Lyft cars clog SF streets, study says,” *San Francisco Chronicle*, 16 October 2018; https://www.sfchronicle.com/business/article/Uber-Lyft-cars-clog-SF-streets-study-says-13309593.php.

[50] Pettersson F., Hiselius L. W., Koglin T., E-commerce and urban planning—Comparing knowledge claims in research and planning practice. Urban Plan. Transp. Res. 6, 1–21 (2018).

[51] San Francisco Center for Economic Development, Tourism Statistics; http://sfced.org/why-san-francisco/facts-figures/tourism/.

