# Supplementary material for: Do transportation network companies decrease or increase congestion?
Source: Sci Adv. 2019 May 8;5(5):eaau2670. doi: 10.1126/sciadv.aau2670 (PMC6506243; doi:10.1126/sciadv.aau2670)
Supplement: http://advances.sciencemag.org/cgi/content/full/5/5/eaau2670/DC1 [file supp_5_5_eaau2670__index.html]

Science Advances | Science Advances

## Supplementary Materials

**The PDF file includes:**

- Supplementary Text
- Fig. S1. Area type map on SF-CHAMP links.
- Table S1. Fixed-effects panel model estimation results only accounting for background traffic.
- Table S2. Network performance metrics by TOD.
- Table S3. Network performance metrics by area type.
- Table S4. Network performance metrics by facility type.
- Legends for Data S1 to S4

Download PDF

**Other Supplementary Material for this manuscript includes the following:**

- Data S1 (.zip format). Supporting data for Fig. 1.
- Data S2 (.zip format). Supporting data for Fig. 2.
- Data S3 (.zip format). Model estimation files.
- Data S4 (.zip format). Model application results and supporting data for Fig. 3.

**Files in this Data Supplement:**

- Adobe PDF - aau2670\_SM.pdf
