# Supplementary material for: Do transportation network companies decrease or increase congestion?
Source: Sci Adv. 2019 May 8;5(5):eaau2670. doi: 10.1126/sciadv.aau2670 (PMC6506243; doi:10.1126/sciadv.aau2670)
Supplement: Download PDF [file aau2670_SM.pdf]

Science Advances
AAAS

advances.sciencemag.org/cgi/content/full/5/5/eaau2670/DC1

Supplementary Materials for

Do transportation network companies decrease or increase congestion?

Gregory D. Erhardt*, Sneha Roy, Drew Cooper, Bhargava Sana, Mei Chen, Joe Castiglione

*Corresponding author. Email: greg.erhardt@uky.edu

Published 8 May 2019, *Sci. Adv.* **5**, eaau2670 (2019)
DOI: 10.1126/sciadv.aau2670

The PDF file includes:

Supplementary Text
Fig. S1. Area type map on SF-CHAMP links.
Table S1. Fixed-effects panel model estimation results only accounting for background traffic.
Table S2. Network performance metrics by TOD.
Table S3. Network performance metrics by area type.
Table S4. Network performance metrics by facility type.
Legends for Data S1 to S4

Other Supplementary Material for this manuscript includes the following:

(available at advances.sciencemag.org/cgi/content/full/5/5/eaau2670/DC1)

Data S1 (.zip format). Supporting data for Fig. 1.
Data S2 (.zip format). Supporting data for Fig. 2.
Data S3 (.zip format). Model estimation files.
Data S4 (.zip format). Model application results and supporting data for Fig. 3.

Supplementary Text

<u>Supplemental Model Estimation Results</u>

As discussed in the main text, one notable variation relates to our hypothesis that TNCs have no effect on traffic congestion. If this were true, we would expect the change in background volume alone to reasonably predict the change in time-implied volume (V_I). **Table S1** shows the estimation results testing this hypothesis. It includes only two of the above parameters: the background volume as estimated by SF-CHAMP, and a scaling factor applied to the Presidio Parkway and Veterans Boulevard. The Presidio parameter can be interpreted as a travel time increase equivalent to reducing the 2010 implied traffic volume by 39%. The background volume is highly correlated with V_I, with a coefficient of 1.78. This suggests that time-implied volumes are increasing by 78% more than SF-CHAMP would predict. It appears that the employment, population and network changes do not fully describe the congestion changes observed during this period, and more terms are needed to do so.

The more complete specification, as reported in the main text, has a better fit and also includes a coefficient on the SF-CHAMP volume that is close to 1. This means that once we account for the effects of TNCs, the change in SF-CHAMP volume reasonably predicts the remaining change.

<u>Supplemental Model Application Results</u>

Table S2. Network performance metrics by TOD. shows the network performance metrics segmented by time-of-day. The results show that the 2016 scenario with TNCs higher VMT, VHT, VHD and BTI80 and lower speeds than the 2016 no TNC scenario throughout the day, including in the AM and PM peak periods.

Table S3. Network performance metrics by area type. shows the network performance metrics segmented by area type. **Figure S1** shows a map of the area types. The metrics show that the effect of TNCs is biggest in the densest area types. For example, in the regional core, the model shows that VHD is 112% higher in 2016 than in 2016, compared to only 13% higher for the no-TNC counterfactual.

Table S4. Network performance metrics by facility type. shows the network performance metrics segmented by facility type. The results show that the effect of TNCs is higher on arterials, collectors and locals than on freeways and ramps.

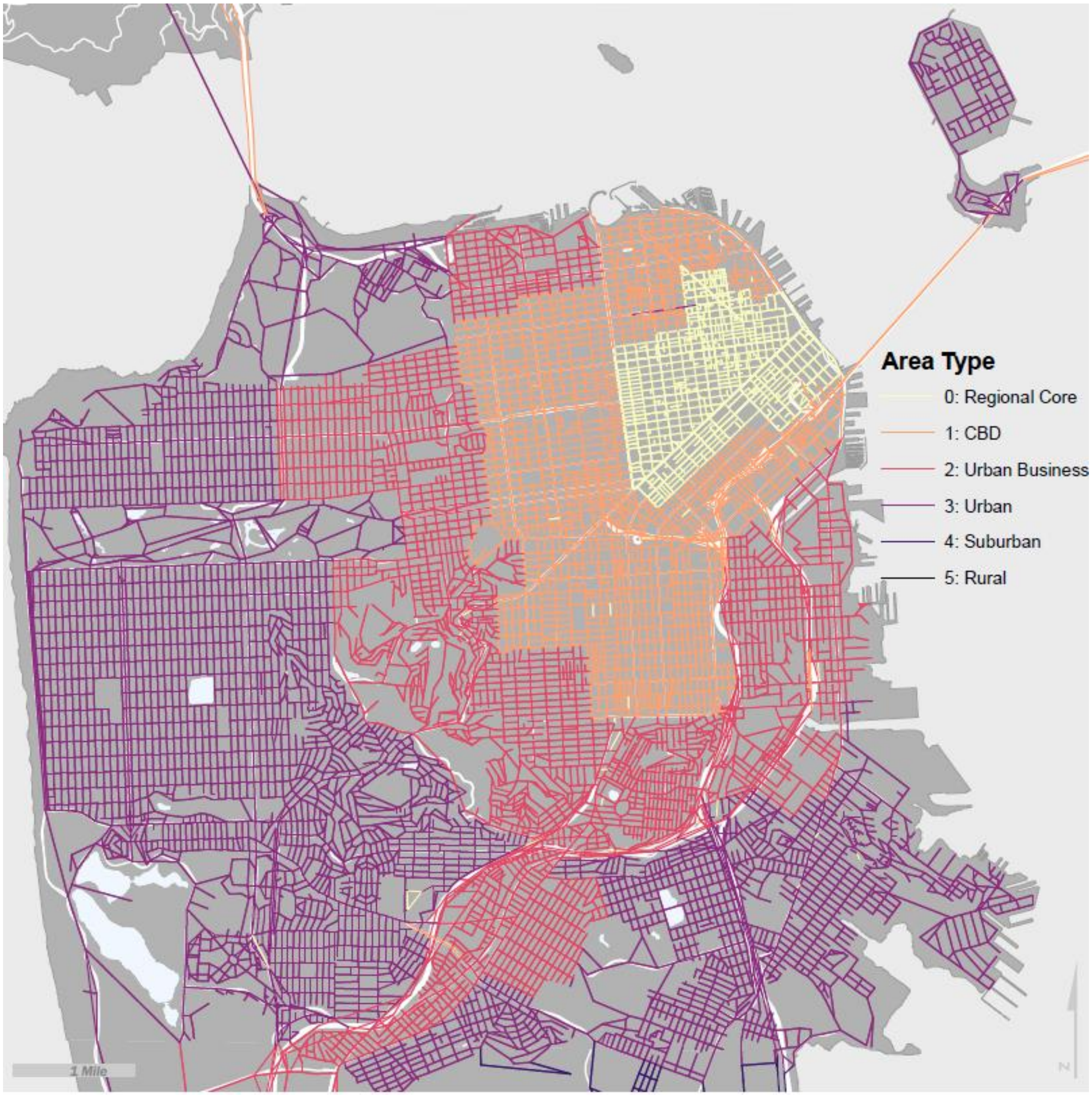

Fig. S1. Area type map on SF-CHAMP links.

Table S1. Fixed-effects panel model estimation results only accounting for background traffic.

**Parameter Estimates**			
Variable	Parameter	Standard Error	T-statistic
SF-CHAMP background volume	1.7816	0.0468	38.052
Presidio Parkway scaling factor	-0.3869	0.0202	-19.144

**Model Statistics**	
Number of Entities	7081
Number of Time Periods	2
R-squared between groups	0.7192
R-squared within groups	0.1941

Table S2. Network performance metrics by TOD.

		Network Performance Metrics								
			Based on Modeled Travel Time				Based on Observed Travel Time			
Time-of-Day	Scenario	Vehicle Miles Traveled	Vehicle Hours Traveled	Vehicle Hours of Delay	Average Speed (mph)	Planning Time Index 80	Vehicle Hours Traveled	Vehicle Hours of Delay	Average Speed (mph)	Planning Time Index 80
6:00 AM-9:00 AM	2010	805,002	32,718	10,180	24.6	1.79	32,955	10,417	24.4	1.95
	2016 No TNC	860,180	36,661	12,509	23.5	1.90	N/A	N/A	N/A	N/A
	2016 with TNC	891,673	40,739	15,467	21.9	2.04	40,651	15,379	21.9	2.33
9:00 AM-3:30 PM	2010	1,848,690	77,735	24,391	23.8	1.74	77,125	23,781	24.0	1.69
	2016 No TNC	1,988,010	88,154	30,587	22.6	1.86	N/A	N/A	N/A	N/A
	2016 with TNC	2,065,117	99,575	39,288	20.7	2.02	101,153	40,867	20.4	2.19
3:30 PM-6:30 PM	2010	1,027,916	49,206	19,485	20.9	2.43	48,137	18,415	21.4	2.32
	2016 No TNC	1,086,243	54,516	23,005	19.9	2.58	N/A	N/A	N/A	N/A
	2016 with TNC	1,126,449	61,819	28,832	18.2	2.80	64,097	31,111	17.6	2.68
6:30 PM-3:00 AM	2010	1,107,141	41,199	9,917	26.9	1.52	41,983	10,700	26.4	1.59
	2016 No TNC	1,196,599	46,103	12,224	26.0	1.59	N/A	N/A	N/A	N/A
	2016 with TNC	1,316,689	58,572	20,473	22.5	1.82	57,306	19,207	23.0	1.86
3:00 AM-6:00 AM	2010	134,700	4,532	890	29.7	1.39	4,487	844	30.0	1.39
	2016 No TNC	149,803	5,208	1,124	28.8	1.43	N/A	N/A	N/A	N/A
	2016 with TNC	159,485	5,689	1,316	28.0	1.47	5,943	1,570	26.8	1.51
Total	2010	4,923,449	205,391	64,863	24.0	1.83	204,686	64,158	24.1	1.83
	2016 No TNC	5,280,836	230,642	79,449	22.9	1.94	N/A	N/A	N/A	N/A
	2016 with TNC	5,559,412	266,393	105,377	20.9	2.12	269,151	108,134	20.7	2.21

		Percent Change from 2010								
			Based on Modeled Travel Time				Based on Observed Travel Time			
Time-of-Day	Scenario	Vehicle Miles Traveled	Vehicle Hours Traveled	Vehicle Hours of Delay	Average Speed (mph)	Planning Time Index 80	Vehicle Hours Traveled	Vehicle Hours of Delay	Average Speed (mph)	Planning Time Index 80
6:00 AM-9:00 AM	2010	0%	0%	0%	0%	0%	0%	0%	0%	0%
	2016 No TNC	7%	12%	23%	-5%	6%	N/A	N/A	N/A	N/A
	2016 with TNC	11%	25%	52%	-11%	14%	23%	48%	-10%	19%
9:00 AM-3:30 PM	2010	0%	0%	0%	0%	0%	0%	0%	0%	0%
	2016 No TNC	8%	13%	25%	-5%	7%	N/A	N/A	N/A	N/A
	2016 with TNC	12%	28%	61%	-13%	16%	31%	72%	-15%	30%
3:30 PM-6:30 PM	2010	0%	0%	0%	0%	0%	0%	0%	0%	0%
	2016 No TNC	6%	11%	18%	-5%	6%	N/A	N/A	N/A	N/A
	2016 with TNC	10%	26%	48%	-13%	15%	33%	69%	-18%	16%
6:30 PM-3:00 AM	2010	0%	0%	0%	0%	0%	0%	0%	0%	0%
	2016 No TNC	8%	12%	23%	-3%	4%	N/A	N/A	N/A	N/A
	2016 with TNC	19%	42%	106%	-16%	19%	36%	79%	-13%	17%
3:00 AM-6:00 AM	2010	0%	0%	0%	0%	0%	0%	0%	0%	0%
	2016 No TNC	11%	15%	26%	-3%	3%	N/A	N/A	N/A	N/A
	2016 with TNC	18%	26%	48%	-6%	5%	32%	86%	-11%	8%
Total	2010	0%	0%	0%	0%	0%	0%	0%	0%	0%
	2016 No TNC	7%	12%	22%	-4%	6%	N/A	N/A	N/A	N/A
	2016 with TNC	13%	30%	62%	-13%	15%	31%	69%	-14%	21%

Table S3. Network performance metrics by area type.

		Network Performance Metrics								
			Based on Modeled Travel Time				Based on Observed Travel Time			
Area Type	Scenario	Vehicle Miles Traveled	Vehicle Hours Traveled	Vehicle Hours of Delay	Average Speed (mph)	Planning Time Index 80	Vehicle Hours Traveled	Vehicle Hours of Delay	Average Speed (mph)	Planning Time Index 80
Regional Core	2010	380,981	28,578	10,214	13.3	2.05	28,529	10,165	13.4	2.08
	2016 No TNC	431,106	34,200	13,516	12.6	2.22	N/A	N/A	N/A	N/A
	2016 with TNC	481,326	46,321	23,202	10.4	2.86	46,652	23,533	10.3	2.87
Central Business District	2010	1,128,774	57,469	19,526	19.6	2.13	56,550	18,608	20.0	2.01
	2016 No TNC	1,213,840	65,430	24,459	18.6	2.28	N/A	N/A	N/A	N/A
	2016 with TNC	1,314,005	78,652	33,814	16.7	2.52	80,327	35,489	16.4	2.67
Urban Business	2010	1,960,197	63,672	18,420	30.8	1.70	63,357	18,105	30.9	1.74
	2016 No TNC	2,107,126	71,715	23,113	29.4	1.81	N/A	N/A	N/A	N/A
	2016 with TNC	2,193,400	78,972	28,060	27.8	1.93	79,536	28,624	27.6	2.08
Urban	2010	1,453,498	55,673	16,704	26.1	1.73	56,249	17,280	25.8	1.76
	2016 No TNC	1,528,763	59,297	18,361	25.8	1.76	N/A	N/A	N/A	N/A
	2016 with TNC	1,570,681	62,448	20,301	25.2	1.82	62,635	20,489	25.1	1.82
Total	2010	4,923,449	205,391	64,863	24.0	1.83	204,686	64,158	24.1	1.83
	2016 No TNC	5,280,836	230,642	79,449	22.9	1.94	N/A	N/A	N/A	N/A
	2016 with TNC	5,559,412	266,393	105,377	20.9	2.12	269,151	108,134	20.7	2.21

		Percent Change from 2010								
			Based on Modeled Travel Time				Based on Observed Travel Time			
Area Type	Scenario	Vehicle Miles Traveled	Vehicle Hours Traveled	Vehicle Hours of Delay	Average Speed (mph)	Planning Time Index 80	Vehicle Hours Traveled	Vehicle Hours of Delay	Average Speed (mph)	Planning Time Index 80
Regional Core	2010	0%	0%	0%	0%	0%	0%	0%	0%	0%
	2016 No TNC	13%	20%	32%	-5%	9%	N/A	N/A	N/A	N/A
	2016 with TNC	26%	62%	127%	-22%	39%	64%	132%	-23%	38%
Central Business District	2010	0%	0%	0%	0%	0%	0%	0%	0%	0%
	2016 No TNC	8%	14%	25%	-6%	7%	N/A	N/A	N/A	N/A
	2016 with TNC	16%	37%	73%	-15%	18%	42%	91%	-18%	33%
Urban Business	2010	0%	0%	0%	0%	0%	0%	0%	0%	0%
	2016 No TNC	7%	13%	25%	-5%	7%	N/A	N/A	N/A	N/A
	2016 with TNC	12%	24%	52%	-10%	14%	26%	58%	-11%	19%
Urban	2010	0%	0%	0%	0%	0%	0%	0%	0%	0%
	2016 No TNC	5%	7%	10%	-1%	2%	N/A	N/A	N/A	N/A
	2016 with TNC	8%	12%	22%	-4%	5%	11%	19%	-3%	4%
Total	2010	0%	0%	0%	0%	0%	0%	0%	0%	0%
	2016 No TNC	7%	12%	22%	-4%	6%	N/A	N/A	N/A	N/A
	2016 with TNC	13%	30%	62%	-13%	15%	31%	69%	-14%	21%

Table S4. Network performance metrics by facility type.

		Network Performance Metrics								
			Based on Modeled Travel Time				Based on Observed Travel Time			
Facility Type	Scenario	Vehicle Miles Traveled	Vehicle Hours Traveled	Vehicle Hours of Delay	Average Speed (mph)	Planning Time Index 80	Vehicle Hours Traveled	Vehicle Hours of Delay	Average Speed (mph)	Planning Time Index 80
Freeways & Ramps	2010	2,201,707	47,332	13,368	46.5	1.77	46,651	12,687	47.2	1.75
	2016 No TNC	2,347,348	51,807	15,602	45.3	1.85	N/A	N/A	N/A	N/A
	2016 with TNC	2,416,922	54,503	17,233	44.3	1.91	56,401	19,132	42.9	2.16
Major Arterials	2010	1,943,506	102,528	33,687	19.0	1.91	102,817	33,976	18.9	1.94
	2016 No TNC	2,102,905	117,620	42,919	17.9	2.05	N/A	N/A	N/A	N/A
	2016 with TNC	2,241,568	139,511	59,512	16.1	2.33	139,680	59,682	16.0	2.29
Minor Arterials	2010	524,855	37,767	12,639	13.9	1.93	37,520	12,392	14.0	1.87
	2016 No TNC	560,389	41,534	14,700	13.5	2.01	N/A	N/A	N/A	N/A
	2016 with TNC	605,131	49,020	20,006	12.3	2.26	49,578	20,564	12.2	2.23
Collectors & Locals	2010	253,381	17,765	5,170	14.3	1.69	17,698	5,103	14.3	1.72
	2016 No TNC	270,194	19,681	6,229	13.7	1.76	N/A	N/A	N/A	N/A
	2016 with TNC	295,791	23,360	8,625	12.7	1.93	23,492	8,757	12.6	1.98
Total	2010	4,923,449	205,391	64,863	24.0	1.83	204,686	64,158	24.1	1.83
	2016 No TNC	5,280,836	230,642	79,449	22.9	1.94	N/A	N/A	N/A	N/A
	2016 with TNC	5,559,412	266,393	105,377	20.9	2.12	269,151	108,134	20.7	2.21

		Percent Change from 2010								
			Based on Modeled Travel Time				Based on Observed Travel Time			
Facility Type	Scenario	Vehicle Miles Traveled	Vehicle Hours Traveled	Vehicle Hours of Delay	Average Speed (mph)	Planning Time Index 80	Vehicle Hours Traveled	Vehicle Hours of Delay	Average Speed (mph)	Planning Time Index 80
Freeways & Ramps	2010	0%	0%	0%	0%	0%	0%	0%	0%	0%
	2016 No TNC	7%	9%	17%	-3%	5%	N/A	N/A	N/A	N/A
	2016 with TNC	10%	15%	29%	-5%	8%	21%	51%	-9%	24%
Major Arterials	2010	0%	0%	0%	0%	0%	0%	0%	0%	0%
	2016 No TNC	8%	15%	27%	-6%	8%	N/A	N/A	N/A	N/A
	2016 with TNC	15%	36%	77%	-15%	22%	36%	76%	-15%	18%
Minor Arterials	2010	0%	0%	0%	0%	0%	0%	0%	0%	0%
	2016 No TNC	7%	10%	16%	-3%	4%	N/A	N/A	N/A	N/A
	2016 with TNC	15%	30%	58%	-11%	17%	32%	66%	-13%	19%
Collectors & Locals	2010	0%	0%	0%	0%	0%	0%	0%	0%	0%
	2016 No TNC	7%	11%	20%	-4%	4%	N/A	N/A	N/A	N/A
	2016 with TNC	17%	31%	67%	-11%	14%	33%	72%	-12%	15%
Total	2010	0%	0%	0%	0%	0%	0%	0%	0%	0%
	2016 No TNC	7%	12%	22%	-4%	6%	N/A	N/A	N/A	N/A
	2016 with TNC	13%	30%	62%	-13%	15%	31%	69%	-14%	21%

Data S1. (separate file) Supporting data for Fig. 1.

Data S2. (separate file) Supporting data for Fig. 2.

Data S3. (separate file) Model estimation files.

Data S4. (separate file) Model application results and supporting data for Fig. 3.
